# Genome-Wide Identification and Expression Analysis of the Alfalfa (*Medicago sativa* L.) U-Box Gene Family in Response to Abiotic Stresses

**DOI:** 10.3390/ijms252212324

**Published:** 2024-11-17

**Authors:** Shuaixian Li, Xiuhua Chen, Meiyan Guo, Xiaoyue Zhu, Wangqi Huang, Changhong Guo, Yongjun Shu

**Affiliations:** 1 Key Laboratory of Molecular Cytogenetics and Genetic Breeding of Heilongjiang Province, College of Life Science and Technology, Harbin Normal University, Harbin 150025, China; hsdlsx1201@stu.hrbnu.edu.cn (S.L.); guomeiyan@163.com (M.G.); zhuxiaoyue2001@126.com (X.Z.); kaku3008@hrbnu.edu.cn (C.G.); 2 International Agriculture Research Institute, Yunnan Academy of Agricultural Sciences, Kunming 650200, China; cxh@yaas.org.cn; 3 National Engineering Research Center for Ornamental Horticulture, Yunnan Flower Breeding Key Laboratory, Flower Research Institute, Yunnan Academy of Agricultural Sciences, Kunming 650200, China

**Keywords:** alfalfa, PUB gene family, abiotic stress, expression pattern, qRT-PCR

## Abstract

E3 ubiquitin ligases known as plant U-box (PUB) proteins regulate a variety of aspects of plant growth, development, and stress response. However, the functions and characteristics of the PUB gene family in alfalfa remain unclear. This work involved a genome-wide examination of the alfalfa U-box E3 ubiquitin ligase gene. In total, 210 members were identified and divided into five categories according to their homology with the members of the U-box gene family in Arabidopsis thaliana. The phylogenetic analysis, conserved motifs, chromosomal localization, promoters, and regulatory networks of this gene were investigated. Chromosomal localization and covariance analyses indicated that the *MsPUB* genes expanded MsPUB gene family members through gene duplication events during evolution. *MsPUB* genes may be involved in the light response, phytohormone response, growth, and development of several biological activities, according to cis-acting element analysis of promoters. In addition, transcriptome analysis and expression analysis by qRT-PCR indicated that most *MsPUB* genes were significantly upregulated under cold stress, drought stress, and salt stress treatments. Among them, *MsPUBS106* and *MsPUBS185* were significantly and positively correlated with cold resistance in alfalfa. *MsPUBS110*, *MsPUBS067, MsPUBS111* and *MsPUB155* were comprehensively involved in drought stress, low temperature, and salt stress resistance. All things considered, these discoveries offer fresh perspectives on the composition, development, and roles of the PUB gene family in alfalfa. They also provide theoretical guidance for further investigations into the mechanisms regulating the development, evolution, and stress tolerance of *MsPUB*.

## 1. Introduction

During the process of development, plants are frequently impacted by many different kinds of abiotic factors, including low temperatures, salinity, and salt. For maintaining the course of ordinary development and growth, plants have developed a series of regulatory processes [1]. Ubiquitination represents a post-translational modification and is often implicated in the growth, development, and stress processes in plants induced by abiotic factors [2]. The ubiquitin–proteasome system (UPS) is a complex system whose core members include ubiquitin (Ub), deubiquitylating enzyme (DUB), ubiquitin-activating enzyme (E1), ubiquitin-conjugating enzyme (E2), ubiquitin ligase enzyme (E3), and 26S proteasome [3]. In order to add ubiquitin to particular target proteins, ubiquitin is first activated by E1, and then it moves to E2 and lastly the E3 ligase [4]. The 26S proteasome typically breaks down target proteins that bind polyubiquitin chains, although monoubiquitinated proteins have the ability to control protein function. E3 ubiquitin ligases are the primary factor that determines the specificity of protein ubiquitination. They work by recognizing and binding to substrates [5]. Depending on how many subunits they have, E3 ubiquitin ligases can be divided into two groups: monomeric and multisubunit. Three types of monomeric E3 ubiquitin ligases can be distinguished: U-box E3 ubiquitin ligases, really interesting new gene (RING), and homology to E6-AP C-terminus (HECT) [6]. In contrast, anaphase-promoting complex/cyclosome (APC/C) E3 ubiquitin ligase complexes and cullin-RING ligase (CRL) are examples of multisubunit E3 ubiquitin ligases [7].

U-box type E3 ubiquitin ligases are widespread in eukaryotes as members of the monomeric group [8]. The 75 amino acid U-box domain of U-box E3 ligase was first found in yeast [9]. In recent years, a growing quantity of plants has been found to contain U-box (PUB) proteins. For example, the PUB gene members are 59 in sorghum [10], 62 in tomato [11], 62 in white pear [12], 65 in cabbage [13], 77 in rice [14], 91 in banana [15], 99 in kale [16], and 121 in moso bamboo [17]. To date, a large number of studies have been conducted to explore the various functions of the U-box gene. In Arabidopsis, *AtPUB18* and *AtPUB19* mutants are more salt-sensitive than the wild type during seed germination, while *AtPUB18* and *AtPUB19* negatively inhibited the ABA-mediated drought stress response [18]. In the apple, the U-box E3 ubiquitin ligase *MdPUB23* reduces cold stress tolerance by degrading the cold stress regulatory protein *MdICE1* [19]. Additionally, it was demonstrated that *MdPUB24* is ethylene-activated and promotes the breakdown of apple chlorophyll [20]. In the strawberry, *FaU-box98* was comprehensively involved in the resistance to ABA, low temperatures, and salt stress. *FaU-box98* and *FaU-box136* were significantly and positively correlated with fruit ripening, while *FaU-box52* was negatively correlated [21]. Furthermore, it has been shown that the peach *PUB* genes are connected to anxin and ABA [22]. These results suggest that *PUB* genes play a critical role in various biological processes, such as fruit ripening and stress responses [23]. However, studies on *PUB* genes in alfalfa are still limited and require further investigation.

Worldwide, alfalfa (*Medicago sativa* L.) is a highly valued feed crop that is abundantly grown. Although it has ecological and economic importance, a variety of factors frequently affect its yield and quality. Therefore, understanding the genetic traits of drought, cold, and salt tolerance in alfalfa is crucial for agricultural development. This study conducted a comprehensive genome-wide analysis of the PUB gene family in alfalfa, including phylogenetic analysis, conserved motifs, chromosomal localization, cis-acting elements, and protein–protein interactions. Additionally, the study explored the responses of alfalfa *PUB* genes under different abiotic stress conditions. These findings offer valuable insights into the role of *PUB* genes in enhancing alfalfa’s stress resilience and provide a foundation for further functional studies of U-box E3 ubiquitin ligase genes in alfalfa.

## 2. Results

### 2.1. Genomic Characterization of Members of the PUB Gene Family in Alfalfa

From the alfalfa genome, 210 *MsPUB* genes were successfully extracted in accordance with the amino acid sequence of the alfalfa PUB gene family. Based on where their chromosomes were located, they were given the names *MsPUBS001* through *MsPUBS210*. Table S1 displays the genomic details of these *MsPUB* genes, such as their names, chromosomal locations, intron counts, and protein lengths (amino acid residues). The longest proteins among these genes are encoded by *MsPUBS003*, *MsPUBS004*, *MsPUBS012*, and *MsPUBS013*; the shortest proteins are encoded by *MsPUBS123*, *MsPUBS126*, and *MsPUBS208*, with 228 amino acid residues. In addition, *MsPUBS206* and *MsPUBS207* had the greatest number of introns (17). Interestingly, they are both members of group VI.

### 2.2. Phylogenetic Analysis of the MsPUBs in Alfalfa

**Figure 1:**
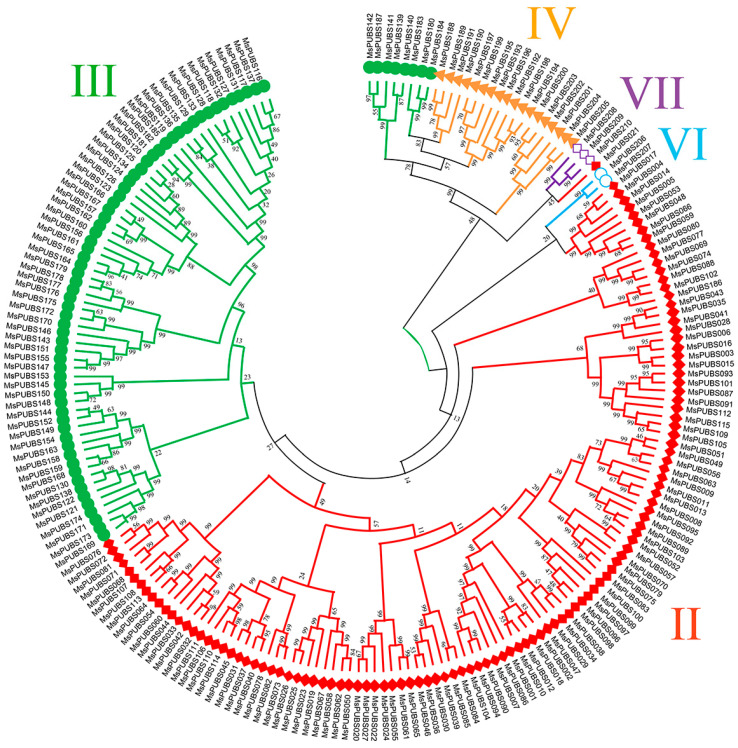
Phylogenetic relationships between *MsPUB* genes. Sequence comparison of 210 MsPUB proteins. Using 1000 bootstrap iterations, the neighbor-joining (NJ) method was used to construct the phylogenetic tree. The five groups of members of the MsPUB gene family are II, III, IV, VI, and VII. Five distinct colors are used to point out the genes in each group.

A phylogenetic tree was constructed using the neighbor-joining (NJ) method to reveal the evolutionary relationships among *MsPUB* genes (Figure 1). Based on the tree topology and consistency with the *AtPUB* classification, the 210 MsPUB gene family members in alfalfa were divided into five major groups: group II, group III, group IV, group VI, and group VII, as shown in Figure 1. Specifically, group II contained the largest number of members, with 115 members, followed by groups III and IV with 72 and 18 members. In addition, group VI has the smallest number of members, with only two members.

### 2.3. Conserved Motif Analysis of the MsPUB Gene Family

**Figure 2:**
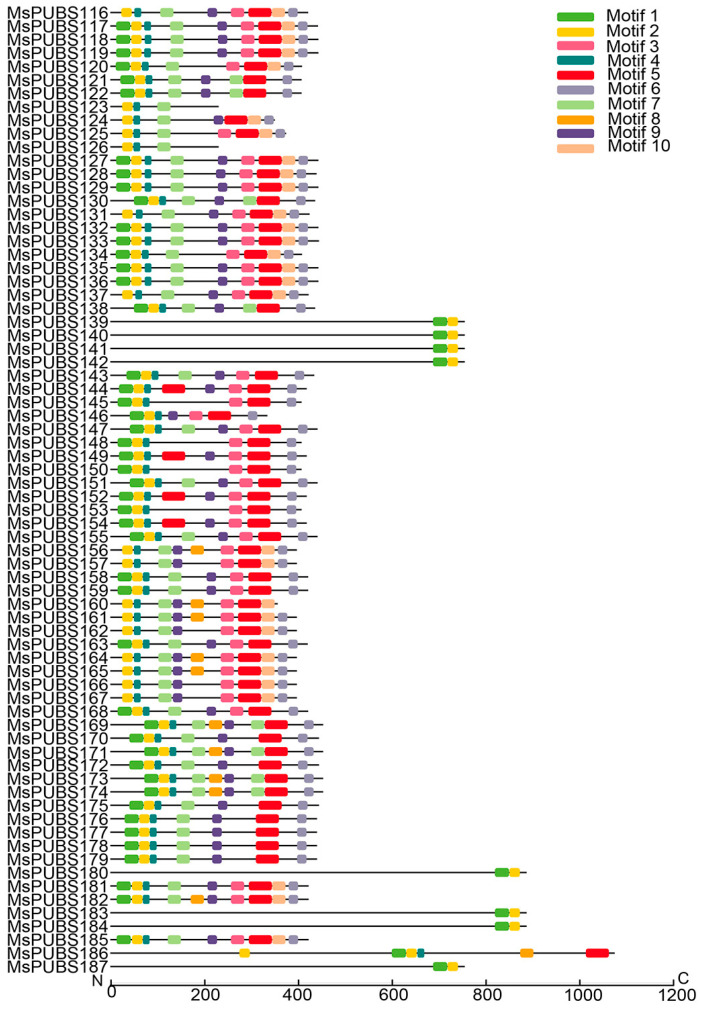
Distribution of conserved motifs in group III of the *MsPUB* genes in alfalfa.

Using the MEME tool (Multiple EM for motif elicitation, version 4.8.1), the conserved motifs and gene structure of the alfalfa *PUB* genes were examined. A total of 10 individual motifs were found in different regions of *MsPUBs*. The findings demonstrated that there were similar conserved motifs in different subfamilies (Figure S1). Detailed information of the 10 conserved motifs can be found in Figure S2. All members of the *MsPUB* genes in group III contain motif 2, and most contain motifs 1, 4, 5, 6, 7 and 9 (Figure 2). Most of the members in group IV contain three motifs, and the members in group VI contain motif 2 and motif 9; the *MsPUB* genes in group VII all contain motif 1. The composition of the conserved motifs in *MsPUB* genes showed that there were obvious motif differences among the five groups, which supported the results of the phylogenetic tree analysis.

### 2.4. Chromosomal Localization Analysis of the MsPUB Genes

**Figure 3:**
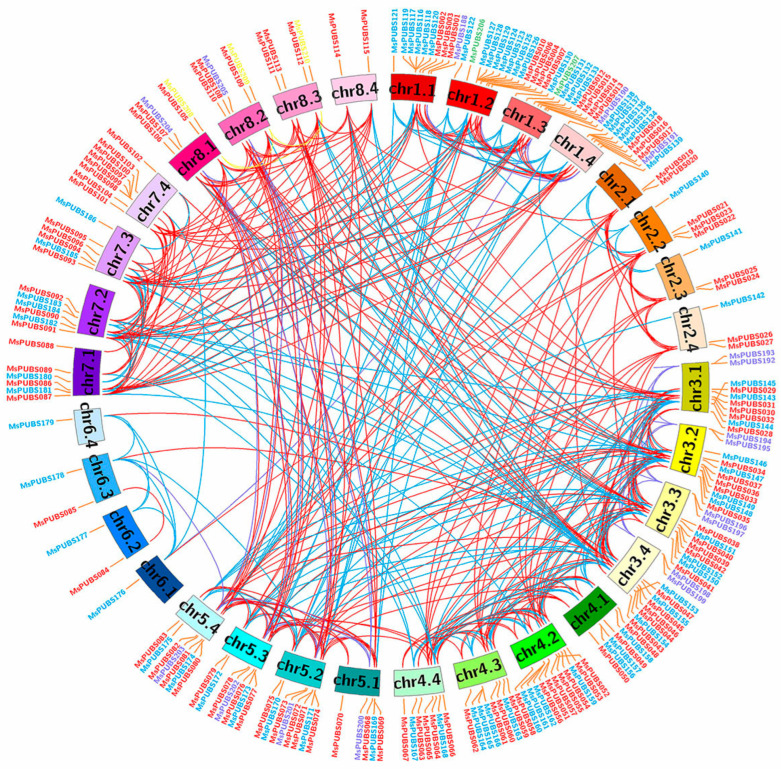
Distribution and replication of *MsPUB* genes. The 210 *MsPUB* genes are located in a ring formed by the 32 chromosomes (chr1.1–chr8.4) of alfalfa. Gene interactions are represented by the colors inside the circles.

The localization map of *MsPUB* genes on chromosomes was constructed using the MCScanX (version python) and Circos software (version 0.69), as shown in Figure 3. Out of the 210 *MsPUB* genes, 209 were successfully mapped onto 32 chromosomes of alfalfa, displaying an uneven distribution. With 18 genes altogether, chr1.2 had the greatest number of *MsPUB* genes. Chr6.1 and chr6.4 had the lowest number of *MsPUB* genes, each with only 1 *MsPUB* gene. The MCScanX program discovered 466 gene duplication occurrences in the alfalfa PUB gene family based on the BLAST results. The findings show that the increase in the MsPUB gene family in the alfalfa genome is largely due to these gene duplication events. The MsPUB gene family has obtained an increased number of members owing to the gene duplication event.

### 2.5. Cis-Acting Elements Analysis of the MsPUB Promoter

**Figure 4:**
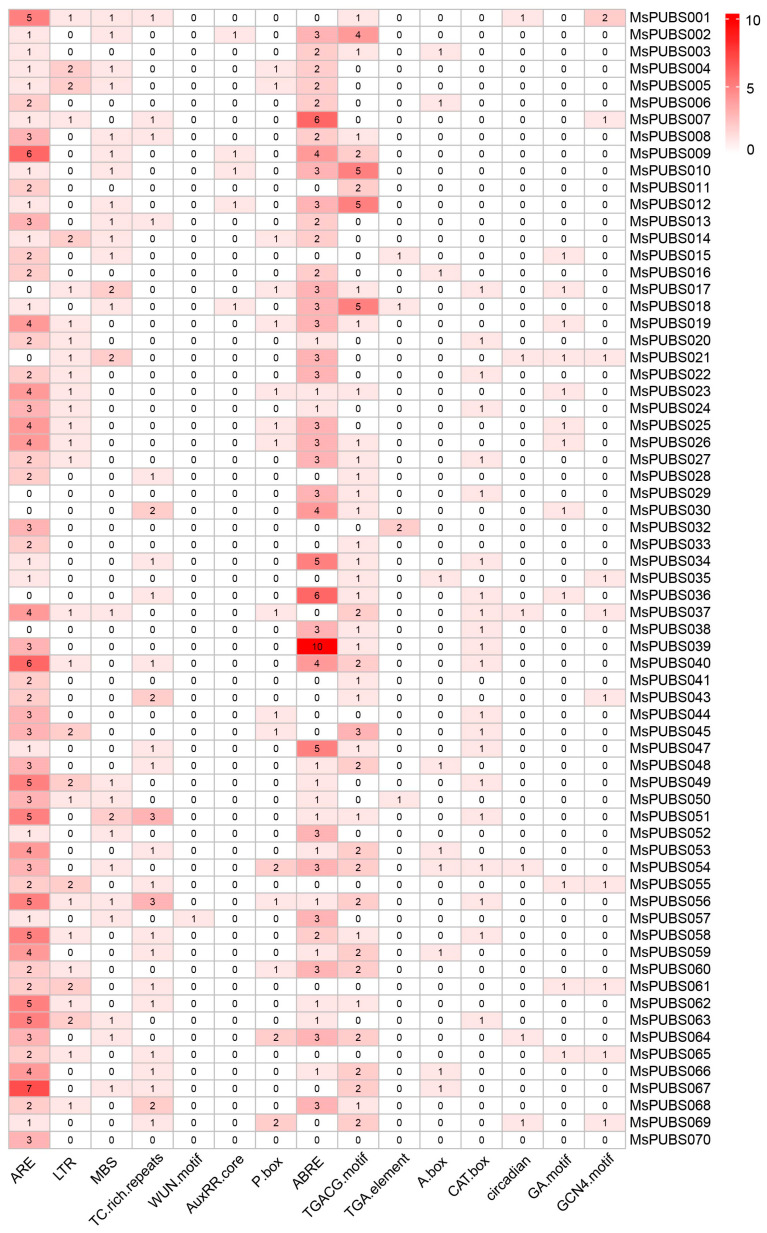
Analysis of cis-acting elements in the promoter regions of the top 70 *MsPUB* genes. The promoter region of *MsPUB* genes contains fifteen cis-acting elements; the more cis-acting elements, the darker the color. The ARE, LTR, MBS, TC-rich repeats, and the WUN motif are related to biotic and abiotic stresses; phytohormone responsiveness is associated with the AuxRR-core, P-box, ABRE, TGACG motif, and TGA element. Plant growth and development are associated with the A-box, CAT-box, circadian, GA motif, and GCN4 motif.

Using the PlantCARE database, cis-acting elements in the promoter region of *MsPUB* genes were investigated in order to learn more about their function. After identifying 83 different types of cis-acting elements, based on the correlation between cis-elements and known regulatory functions, including plant growth and development, hormone response, and abiotic and biotic stress, 15 cis-acting elements were selected for further analysis [24]. The cis-acting elements of all members of the MsPUB gene family are shown in Figure S3. The cis-acting elements in the promoter regions of the first 70 *Ms PUB* genes are shown in Figure 4. In the category of growth and developmental elements, we identified the light-responsive regulatory element A-box, the circadian regulatory element, the meristematic tissue expression element CAT-box, the endosperm expression element GCN4 motif, and the light-responsive element involved in GA motif. Furthermore, the plant response to biotic stress is mediated by ARE, TC-rich repeats, and the WUN motif, which are found in a total of 482,104 and 11 cis-acting elements, respectively. There were 100 low-temperature-responsive cis-element LTRs and 123 drought-induced element MBSs among the abiotic stress-response elements. Among the hormone-response elements, there were 393 abscisic acid-related elements (ABRE) and 508 MeJA-related elements (255 TGACG motifs, 253 CGTCA motifs), as well as 76 gibberellin-responsive P-box motifs, 32 growth hormone-related AuxRR-core elements, and 14 TGA elements. The results presented reveal that *MsPUB* genes might be actively involved in regulating the responses of phytohormones and different stressors.

### 2.6. Genetic Regulatory Network Analysis of MsPUB Genes

**Figure 5:**
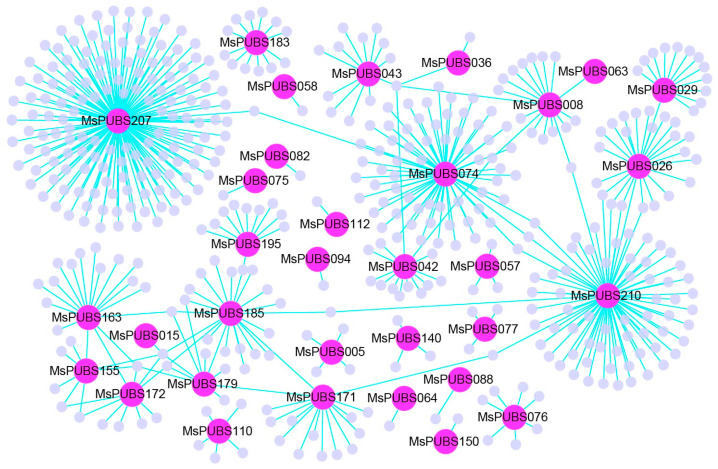
Analysis of the interactions between *MsPUB* genes in alfalfa using gene regulatory networks. The gene regulatory network (GRN) of *MsPUB* genes and their interactions was built using Cytoscape and was based on relationships between genes in Arabidopsis thaliana. The cyan line shows the interaction of alfalfa, the pink nodes relate to *MsPUB* genes, and the purple nodes correspond to genes interacting with *MsPUB* genes.

**Figure 6:**
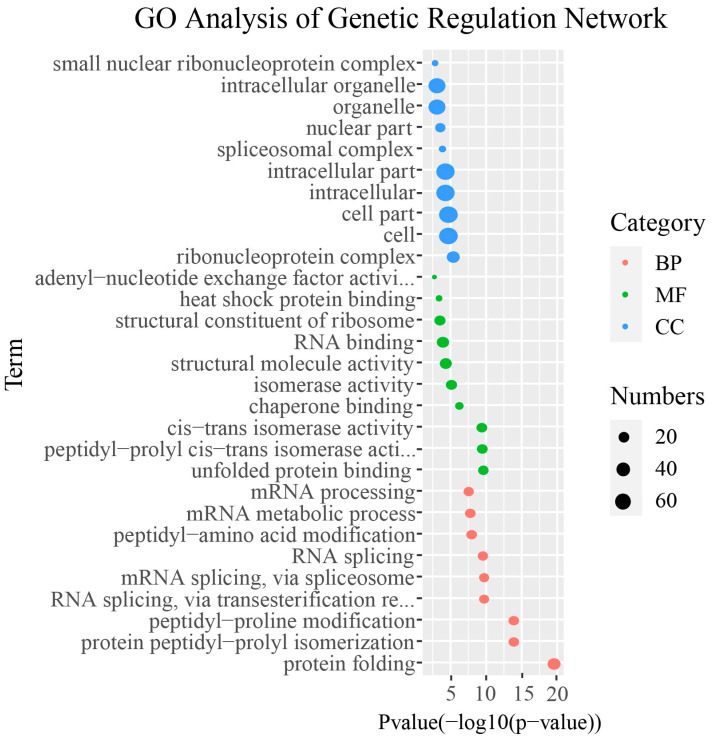
A review of the genes that interact with *MsPUB* genes and their gene ontology enrichment. The GO terms for molecular functions (MFs), cellular components (CCs), and biological processes (BPs) are shown as red, green, and blue dots, respectively. The GO term is displayed on the *Y*-axis, while the *X*-axis displays the *p*-value of the topGO enrichment analysis with −log 10 transformation or −log 10 (*p*). The number of genes involved in the GO keywords is reflected in the size of the circles.

The gene regulatory network (GRN) is a complex network system composed of a series of genes and their regulatory factors. As shown in Figure 5, the GRNs of *MsPUBs* and their interacting genes contain a total of 493 genes and 498 interactions. From the GRN, the majority of *MsPUBs* were discovered to interact with many functional genes. For example, *MsPUBS207* interacts with 149 genes, *MsPUBS210* interacts with 72 genes, *MsPUBS074* interacts with 65 genes, and *MsPUBS026* interacts with 24 genes. GO enrichment analysis was performed using R software (version 4.3.3) with the topGO program package (version 2.38.31). In Figure 6, a large number of MsPUB genes are distributed within cells and organelles. In addition, these functional genes are mainly focused on regulating ribosomal protein assembly and ribosomal RNA splicing.

### 2.7. Expression Analysis of Alfalfa MsPUBs Under Abiotic Stresses

**Figure 7:**
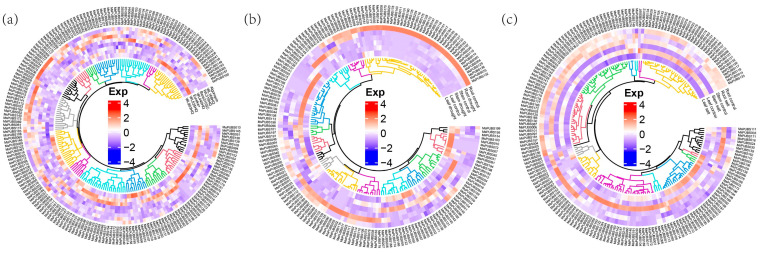
Expression profiles of *MsPUB* under stress conditions. (**a**) Eight varieties of alfalfa were analyzed under cold stress. (**b**) Under drought stress, the performance of Wilson and PI467895 alfalfa was studied in three tissues of the root, stem, and leaf. (**c**) Under salt stress, the performance of Wilson and PI467895 alfalfa in three tissues of rhizome leaves was investigated. The R platform was used to present the mean expression levels (FPKM values), which were measured using Salmon software (version 0.12.0).

To learn more about how *MsPUB* responds to abiotic stressors through engagement, the RNA-seq of alfalfa under different stresses was downloaded for further expression analysis. In Figure 7a, under cold stress treatment, the expression levels of *MsPUBS023*, *MsPUBS113*, *MsPUBS185*, and *MsPUBS205* were upregulated. *MsPUB147* exhibited the highest expression levels, indicating a stronger response to cold stress compared to other genes. In Figure 7b, *MsPUB* gene expression was upregulated in the tissues of the roots, stems, and leaves when the drought stress treatment was applied; the expression level in the roots was remarkably higher. For instance, this was the case in the *MsPUBS022*, *MsPUBS036*, and *MsPUBS090* genes. In addition, *MsPUBS172* showed the highest expression under salt stress, suggesting it is more responsive to salt stress than the other genes. In Figure 7c, when the salt stress treatment was applied to the root, stem, and leaves, the majority of *MsPUB* genes had elevated expression levels, such as *MsPUBS032*, *MsPUBS059*, and so on. Furthermore, *MsPUB183* also had the highest expression under salt stress, indicating its greater responsiveness to salt stress relative to the other genes. These results demonstrated the important contribution of the MsPUB gene family to abiotic stress response.

### 2.8. qRT-PCR Verification of MsPUB Gene Expression Under Abiotic Stress

**Figure 8:**
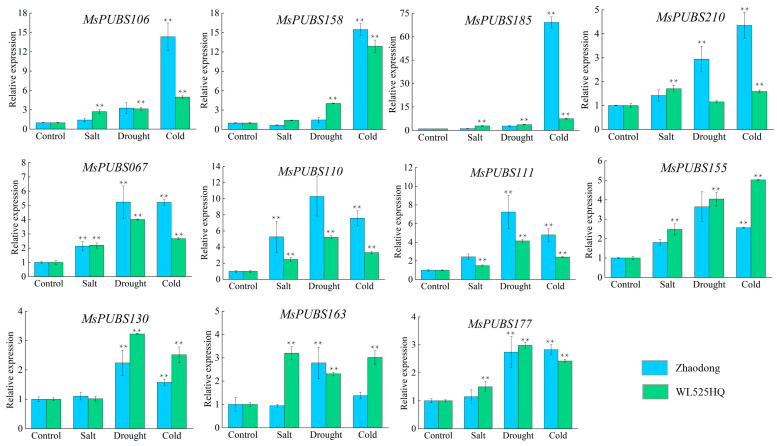
qRT-PCR analysis of *MsPUBs* under abiotic stress. qRT-PCR analysis of *MsPUBs* under salt, drought, and cold stresses. Eleven abiotic stress-responsive genes were selected for qRT-PCR experiments. The *X*-axis represents control, salt treatment, drought treatment, and cold treatment. The *Y*-axis represents the relative expression level of *MsPUB* genes. The relative expression was calculated using the 2^−ΔΔCT^ method, with the expression level of the “control” set to 1. *GAPDH* was used as the internal control. The blue color represents *Zhaodong* and the green color represents WL525HQ. Statistical significance was determined using the *t*-test (** *p* < 0.01). Asterisks indicate significant differences.

Eleven *MsPUB* genes were chosen for quantitative reverse transcription–polymerase chain reaction (qRT-PCR) analyses in order to confirm the genes’ quick sensitivity to abiotic stress. *GAPDH* was used as an internal reference, and each experiment was repeated three times (Table S2). The results showed that the expression levels of 11 *MsPUB* genes were significantly changed under cold stress, drought stress, and salt stress, as shown in Figure 8. Among them, the transcript levels of *MsPUBS106*, *MsPUBS158*, *MsPUBS185*, and *MsPUBS210* showed a significant upward trend after cold stress treatments and were more obvious in the Zhaodong variety. In addition, *MsPUBS177* and *MsPUBS130* were significantly upregulated in expression under drought stress treatment, compared with other abiotic stresses and the control. It is significant that in conditions of cold, drought, and salt stress, *MsPUBS155*, *MsPUBS110*, *MsPUBS111*, and *MsPUBS067* were considerably upregulated. These four *MsPUB* genes have a broad spectrum in the abiotic stress response of alfalfa. Overall, *MsPUB* genes were clearly engaged in alfalfa’s response to abiotic stressors, as shown by the generally similar RNA-seq and qRT-PCR data analysis results.

## 3. Discussion

Within the E3 ubiquitin ligase family, plants include a substantial amount of U-box genes [25]. So far, extensive research has been carried out on the traits and roles of the PUB gene family for several plant species, such as cabbage, rice, and white pears [12,13,14]. In wheat, *TaPUB1* increases the plant’s resilience to salt and drought, whereas *TaPUB4* influences the anther’s metabolism of sucrose and starch, which in turn controls the formation of pollen [26,27]. Alfalfa, as an important perennial herbaceous plant, has both economic and ecological values [28]. In this investigation, we discovered 210 *PUB* genes in the genome of alfalfa (Table S1). The phylogenetic analysis of *MsPUB* genes was conducted using the Arabidopsis PUB gene family as a reference. It was visible from the phylogenetic tree analysis that the 210 *MsPUBs* were divided into five categories (Figure 1). The group I *PUB* genes present in the Arabidopsis U-box gene family are missing in alfalfa, which may be due to the fact that Arabidopsis only has one member in the group I PUB gene family. During the evolutionary divergence of the PUB gene families between Arabidopsis and alfalfa, this specific gene group may have been lost or altered in alfalfa. Alfalfa may have compensated for this loss through other *PUB* genes or alternative mechanisms, potentially resulting in different stress regulation pathways. Furthermore, the development of this family inside the alfalfa genome has been shown by the significantly higher number of *PUB* genes in the genome compared to the banana [15] and pear [12], indicating the expansion of this family in the alfalfa genome. The MsPUB gene family contained 466 repeated occurrences, according to the results of the collinearity analysis (Figure 3). This duplication appears to have resulted from a recent genome-wide duplication event in the alfalfa genome, revealing a non-random conserved duplication pattern that is comparatively frequent among species [29]. We conducted further analysis on the types of gene duplication and found that duplication events in the PUB gene family in alfalfa are primarily segmental duplications. Segmental duplications typically involve multiple genes, which can significantly increase the number of gene family members, consistent with our observation of 210 MsPUB gene family members (Table S1). Because these segmental duplication gene copies are distributed across different genomic regions, they are likely influenced by distinct regulatory elements, gradually leading to functional diversification and adaptation to various environmental pressures [30]. Therefore, we propose that this duplication pattern may have driven the adaptive evolution of the PUB gene family in alfalfa, providing an important genetic foundation for environmental resilience and enhanced survival.

Many *U-box* genes influence drought tolerance in plants through diverse mechanisms, according to previous studies. For instance, *OsPUB67*, *AtPUB11*, *AtPUB18*, and *AtPUB19* are ABA-dependently engaged in drought tolerance [18,31,32]. *GmPUB6* overexpressing plants showed significantly upregulated drought-responsive genes, suggesting that *GmPUB6* mediates ABA signaling pathways and osmotic stress to improve plant drought tolerance [33]. We also found a large number of abiotic response elements in the alfalfa PUB promoter region, particularly those associated with abiotic stress, ABA, JA, and GA responses (Figure 4). In the identification of hormone-related elements, the ABRE element is associated with the abscisic acid (ABA) signaling pathway, the P-box element is linked to the gibberellin (GA) signaling pathway, and the AuxRR-core element is involved in the auxin signaling pathway [34]. These three elements primarily regulate plant defense responses, especially in response to abiotic stresses such as water stress, drought, and salt stress. This suggests that *MsPUB* genes may respond to abiotic stresses by modulating hormonal signaling pathways. We speculate that the *PUB* genes may induce alfalfa hormone signal transduction in response to abiotic stress. Additionally, GO analysis shows that *MsPUB* genes are primarily associated with the regulation of ribosomal protein assembly and ribosomal RNA splicing. These functions may be linked to the ability of U-box type E3 ubiquitin ligases to respond to abiotic stress through protein degradation pathways mediated by ubiquitination (Figure 6).

Abiotic stressors like cold, salinity, and drought are harmful for plant growth and development, which reduces global food yield significantly [12]. In order to survive and keep developing, plants have developed complex systems to react quickly to changes in their surroundings. While *AtPUB25* and *AtPUB26* are involved in the reaction of plants to low temperatures, *AtPUB22* and *AtPUB23* are negative regulators that mediate the response to drought [35]. As a consequence of the study’s findings, the majority of genes, including *MsPUBS023*, *MsPUBS113*, *MsPUBS185*, and *MsPUBS205*, had their expression levels elevated under cold stress. Certain *MsPUB* genes exhibit stronger expression under specific stress conditions. For instance, *MsPUBS147* showed the highest expression under cold stress, suggesting that it may play a key role in cold stress response. Similarly, *MsPUBS172* exhibited higher expression under salt stress, while *MsPUBS183* showed elevated expression under drought stress, indicating that these genes may be more sensitive to salt or drought stress compared to others. These differential expression patterns suggest that these *MsPUB* genes could serve as potential markers for specific stress responses, such as cold or salt tolerance, and may contribute to the development of stress-resistant varieties. However, further functional studies are needed to confirm their roles in stress tolerance mechanisms (Figure 7). At the same time, a cis-acting element LTR implicated in low temperature responsiveness exists in the promoter region of the *MsPUB* genes (Figure 4). In addition, the expression levels of *MsPUBS022*, *MsPUBS036*, and *MsPUBS090* were significantly higher in roots compared to stems and leaves, suggesting that these genes have a tissue-specific function, likely tied to processes that are particularly important in root development or response to environmental stress. Such tissue-specific expression patterns are common for genes involved in stress resistance, nutrient uptake, or developmental pathways, with roots playing a key role in sensing and adapting to soil conditions like drought, salinity, or pathogen presence. It also aligns with the earlier outcomes. During the early developmental stages of banana fruit tissues, the MaU-box gene family was the most expressed in roots [12]. Similarly, in foxtail millet, the expression of *SiPUB* genes varied significantly across different tissues, with the majority showing high expression levels in the roots. These observations suggest that *PUB* genes play a crucial role in root development as well as other growth and developmental processes in crops [36]. The high responsiveness of the *PUB* genes to abiotic stress in alfalfa was further verified by qRT-PCR (Figure 8). In conclusion, our systematic identification of the alfalfa PUB gene family laid the groundwork for next studies aimed at functionally validating the PUB gene family.

## 4. Materials and Methods

### 4.1. Genome Identifcation of MsPUBs in Alfalfa

The Pfam database was used to retrieve the Hidden Markov Model (HMM) file (PF04564) corresponding to the U-box domain so as to identify the members of the alfalfa PUB gene family [17]. The members of the alfalfa genome with U-box domains were found using HMMER (version 3.1) The expected (e) cutoff of 0.01 was used to identify and validate the domains of the PUB proteins. The alfalfa genome was searched using the Arabidopsis U-box protein (PUB) sequence as a BLAST query sequence, with an E-value threshold of 1 × 10^−5^ and coverage set at 80%. The PUB sequence was downloaded from the TAIR database (https://www.arabidopsis.org) (accessed on 15 January 2024) [37]. All possible *PUB* genes were identified by their genomic location, length of protein, and number of introns, which were retrieved from the alfalfa genome. Subsequently, based on how similar these *MsPUB* genes were to Arabidopsis *PUB* genes, they were divided into a number of categories [9].

### 4.2. Phylogenetic Analysis of MsPUBs in Alfalfa

For the evolutionary analysis of *MsPUB* genes, a number of alignments of putative alfalfa PUB protein sequences were performed using MUSCLE (version 5.1.0)with default settings [38]. Phylogenetic tree construction was conducted using the neighbor-joining (NJ) approach with 1000 bootstrap repetitions and MEGA 11.0 software [39].

### 4.3. Analysis of the Distribution of Motif Composition in MsPUB Proteins in Alfalfa

The MEME program was used to search for conserved motifs in the sequences of alfalfa PUB proteins, and the following settings were used to look for conserved motifs in MsPUB protein sequencing: up to ten motifs; arbitrary number of repetitions of a single motif; and minimum and maximum widths of six and fifty [40]. TBtools (version 2.102) is used to present all results [41].

### 4.4. Chromosomal Localization Analysis of MsPUBs in Alfalfa

Using BLASTP software (version 2.9.0+), all alfalfa proteins were compared to one another. To learn more about the specific locations of the chromosomes that make up the PUB gene family, the genome file’s alfalfa annotation was utilized. Based on this information gene, duplicates were identified and characterized through MCSanX software (Version python) using default parameters [42]. The chromosomal localization of *PUB* genes in alfalfa was mapped by Circos and the TBtools software (version 2.102) and revealed the syntenic relationships of *MsPUB* genes [43].

### 4.5. Cis-Acting Element Analysis of MsPUBs in Alfalfa

The 2000 bp genomic sequence upstream of the start codon of the *MsPUB* genes was taken from the genomic sequence in order to study cis-acting elements in the promoter of the *MsPUB* genes. Utilizing the PlantCARE database, cis-acting elements were analyzed [44].

### 4.6. Analysis of the Gene Regulatory Network of the MsPUB Gene Family in Alfalfa

The AraNet database (V2) provided information on the Arabidopsis gene regulatory network (GRN) [45]. All proteins of alfalfa and *Arabidopsis* were retrieved by BLAST to establish homologous relationships. The homologs of *Arabidopsis* or alfalfa genes were identified among the hits with the highest scores, which were obtained by performing these searches at an E-value threshold of 1 × 10^−5^. Based on the two BLAST results, the procedure produced homologous gene pairs. After that, an alfalfa-like GRN was created utilizing the Arabidopsis GRN and the data from these homologous gene pairs. Using Cytoscape software (Version 3.9.1), the MsPUB gene-containing alfalfa subnetwork was located and investigated and the results were displayed [46]. Using topGO (Version 2.38.31), gene ontology (GO) analysis of enrichment was carried out on the subnetwork, with an acceptable threshold of 0.05, which identified extremely rich phrases using GRN (from the AraNet database (V2) functions and showed representations of the most significant terms, as the software protocol specifies.

### 4.7. Analysis of MsPUBs Gene Expression Using RNA-Seq Data

The NCBI database provided the alfalfa RNA-seq datasets. RNA-seq data were used to examine the responses of eight alfalfa cultivars to cold stress (accession number: PRJNA780579) [47] and three different tissues of Wilson and PI467895 under drought stress and salt stress response (accession number: PRJNA667169) [48]. Salmon software (version 0.12.0) was utilized to compute the expression levels of every gene, and the transcript sequences of the alfalfa genome were compared with the RNA-seq data [49]. R software (version 4.3.3) was used to cluster and display the data [47].

### 4.8. Plant Materials and Stress Treatments

Alfalfa seeds from Zhaodong and WL525HQ were germinated and then transplanted into a mixture of perlite and sand in a 3:1 volume ratio [50]. The growth temperature was 24 °C, the illumination time was 16 h, and the darkness was 8 h. Irrigation with Hoagland nutrient solution was performed every 2 days. After 45 days of growth, all seedlings were divided into four groups. For both the salt and drought treatments, two groups of seedlings received irrigations using 20% PEG-6000 and 100 mM NaCl nutritional solution. The other group of seedlings was cooled in a refrigerator at 4 °C. The final group of seedlings received regular nutrient solution irrigation without any additional substances, serving as the control. For all four groups, alfalfa leaves were collected after treatments under normal light conditions for 1 h, 3 h, and 4 h. Leaves at three different time points were collected and mixed. After being instantly frozen in liquid nitrogen, every sample was kept at −80 °C in order to facilitate further RNA extraction.

### 4.9. QRT-PCR Analysis

Total RNA was extracted from alfalfa leaves subjected to control, low-temperature stress (4 °C), drought stress, and salt stress using the RNApure Plant Kit (Tiangen, Beijing, China) according to the manufacturer’s instructions. DNase I from the kit was used to remove genomic DNA during RNA extraction. The integrity of RNA was confirmed by gel electrophoresis, and the RNA was stored at −80 °C until further use. For cDNA synthesis, 1 µg of total RNA was reverse-transcribed using the PrimeScript RT Kit (Toyobo, Shanghai, China) according to the manufacturer’s protocol. The synthesized cDNA was stored at −20 °C for subsequent quantitative real-time PCR (qRT-PCR) analysis. According to the nucleotide sequence of PUB family genes, the Primer 5.0 software was used to design 11 pairs of particular primers (Table S2). SYBR PreMix Ex TaqTMII (TOYOBO, Shanghai, China) was used for qRT-PCR. The qRT-PCR program was set as follows: 95 °C for 2 min, followed by 40 cycles of 95 °C for 30 s, 55 °C for 30 s, and 72 °C for 1 min. Each experiment was performed in triplicate. A melting curve analysis was conducted at the end of each run to verify the specificity of the amplification. The corresponding expression level of *MsPUBs* was computed using the 2^−∆∆CT^ technique [51].

## 5. Conclusions

This study found 210 *PUB* genes in alfalfa and performed several analyses on them, including GO annotations, qRT-PCR assays, cis-acting elements, chromosomal localization, gene expression analyses, and phylogenetic and motif composition. We discovered that there were gene duplication events across the evolution of the PUB gene family. Meanwhile, ABRE, P-box, TGACG motif elements, MBS, LTR, and other abiotic stress and parts of the hormone response are abundantly present in the promoter regions of *MsPUBs*. In alfalfa, *MsPUB* genes showed a strong reaction to abiotic stress, suggesting that *MsPUB* genes play a vital role in regulating how plants respond to various kinds of abiotic challenges. The findings of this work serve as a foundation for more research into the role of the PUB gene family in alfalfa under abiotic stressors.

## Data Availability

The datasets presented in this study can be found in Supplementary Materials.

## Supplementary Materials

The following supporting information can be downloaded at: https://www.mdpi.com/article/10.3390/ijms252212324/s1.
