# Supplementary material for: Genome-Wide Identification and Expression Analysis of the Alfalfa (Medicago sativa L.) U-Box Gene Family in Response to Abiotic Stresses
Source: Int J Mol Sci. 2024 Nov 17;25(22):12324. doi: 10.3390/ijms252212324 (PMC11595061; doi:10.3390/ijms252212324)
Supplement: Supplementary file 1 [file ijms-25-12324-s001.zip › Suppl. Figure captions.pdf]

Figure S1: Conserved motif analysis of 210 *MsPUB* genes.
Figure S2: Information of 10 conserved motifs.
Figure S3: Analysis of cis-acting elements of 210 MsPUB members.
