# Supplementary material for: Genome-Wide Identification and Expression Analysis of the Alfalfa (Medicago sativa L.) U-Box Gene Family in Response to Abiotic Stresses
Source: Int J Mol Sci. 2024 Nov 17;25(22):12324. doi: 10.3390/ijms252212324 (PMC11595061; doi:10.3390/ijms252212324)
Supplement: Supplementary file 1 [file ijms-25-12324-s001.zip › Table S1.pdf]

**Table S1.** Summary of *MsPUB* genes identified in alfalfa

| Name | Gene ID | Chromosomal locations | Group | Intron | Length(aa) |
|---|---|---|---|---|---|
| *MsPUBS001* | MS.gene005886 | chr1.1:67901714-67904057 | II | 3 | 411 |
| *MsPUBS002* | MS.gene036072 | chr1.1:51944855-51949701 | II | 4 | 726 |
| *MsPUBS003* | MS.gene062248 | chr1.1:57259261-57262720 | II | 2 | 1013 |
| *MsPUBS004* | MS.gene005714 | chr1.2:67523533-67530332 | II | 15 | 1489 |
| *MsPUBS005* | MS.gene050173 | chr1.2:67553591-67565400 | II | 15 | 1489 |
| *MsPUBS006* | MS.gene07411 | chr1.2:64102929-64106389 | II | 2 | 1013 |
| *MsPUBS007* | MS.gene34060 | chr1.2:73605897-73609686 | II | 5 | 733 |
| *MsPUBS008* | MS.gene34076 | chr1.2:74259536-74264135 | II | 3 | 630 |
| *MsPUBS009* | MS.gene34077 | chr1.2:74273381-74277279 | II | 3 | 376 |
| *MsPUBS010* | MS.gene50646 | chr1.2:53087326-53092162 | II | 4 | 726 |
| *MsPUBS011* | MS.gene51233 | chr1.2:74304675-74306255 | II | 3 | 454 |
| *MsPUBS012* | MS.gene000356 | chr1.3:49439102-49443945 | II | 4 | 726 |
| *MsPUBS013* | MS.gene005921 | chr1.3:65542663-65546453 | II | 3 | 630 |
| *MsPUBS014* | MS.gene21595 | chr1.3:63150873-63157673 | II | 15 | 1489 |
| *MsPUBS015* | MS.gene85583 | chr1.3:54887399-54890807 | II | 2 | 1013 |
| *MsPUBS016* | MS.gene004887 | chr1.4:62698264-62701728 | II | 2 | 1013 |
| *MsPUBS017* | MS.gene21370 | chr1.4:71588031-71594834 | II | 15 | 1489 |
| *MsPUBS018* | MS.gene79291 | chr1.4:56801531-56806377 | II | 4 | 726 |
| *MsPUBS019* | MS.gene002627 | chr2.1:68988163-68990319 | II | 0 | 718 |
| *MsPUBS020* | MS.gene02272 | chr2.1:72697820-72698971 | II | 0 | 383 |
| *MsPUBS021* | MS.gene003075 | chr2.2:60865723-60871404 | II | 7 | 350 |
| *MsPUBS022* | MS.gene01302 | chr2.2:70614197-70615348 | II | 0 | 383 |
| *MsPUBS023* | MS.gene01612 | chr2.2:66910432-66912588 | II | 0 | 718 |
| *MsPUBS024* | MS.gene002323 | chr2.3:72512155-72513298 | II | 1 | 348 |
| *MsPUBS025* | MS.gene03102 | chr2.3:69000466-69002622 | II | 0 | 718 |
| *MsPUBS026* | MS.gene004304 | chr2.4:68190596-68192752 | II | 0 | 718 |
| *MsPUBS027* | MS.gene01715 | chr2.4:72447669-72448820 | II | 0 | 383 |
| *MsPUBS028* | MS.gene070001 | chr3.1:91524168-91527901 | II | 2 | 814 |
| *MsPUBS029* | MS.gene41564 | chr3.1:50483377-50488736 | II | 3 | 815 |
| *MsPUBS030* | MS.gene70600 | chr3.1:64268812-64270392 | II | 0 | 526 |
| *MsPUBS031* | MS.gene70612 | chr3.1:64164888-64166513 | II | 0 | 541 |
| *MsPUBS032* | MS.gene78985 | chr3.1:68356888-68360453 | II | 4 | 765 |
| *MsPUBS033* | MS.gene045255 | chr3.2:73556068-73559195 | II | 3 | 748 |
| *MsPUBS034* | MS.gene049489 | chr3.2:56853241-56858965 | II | 5 | 867 |
| *MsPUBS035* | MS.gene064429 | chr3.2:91486069-91489791 | II | 2 | 814 |
| *MsPUBS036* | MS.gene25928 | chr3.2:69212618-69214198 | II | 0 | 526 |
| *MsPUBS037* | MS.gene25941 | chr3.2:69064784-69066823 | II | 0 | 679 |
| *MsPUBS038* | MS.gene008561 | chr3.3:54157882-54163630 | II | 5 | 858 |
| *MsPUBS039* | MS.gene022271 | chr3.3:66395463-66397052 | II | 0 | 529 |
| *MsPUBS040* | MS.gene022282 | chr3.3:66300801-66302840 | II | 0 | 679 |
| *MsPUBS041* | MS.gene064856 | chr3.3:94484354-94488087 | II | 2 | 814 |

**Table S1. (continued)**

| | | | | | |
|---|---|---|---|---|---|
| *MsPUBS042* | MS.gene75591 | chr3.3:71421426-71425002 | II | 4 | 766 |
| *MsPUBS043* | MS.gene014327 | chr3.4:98835497-98839230 | II | 2 | 814 |
| *MsPUBS044* | MS.gene066823 | chr3.4:79936434-79940010 | II | 4 | 766 |
| *MsPUBS045* | MS.gene074370 | chr3.4:74904998-74906623 | II | 0 | 541 |
| *MsPUBS046* | MS.gene074381 | chr3.4:74995261-74996841 | II | 0 | 526 |
| *MsPUBS047* | MS.gene38510 | chr3.4:61847280-61855219 | II | 3 | 810 |
| *MsPUBS048* | MS.gene049106 | chr4.1:2832434-2840388 | II | 14 | 1337 |
| *MsPUBS049* | MS.gene41180 | chr4.1:36725959-36730423 | II | 3 | 662 |
| *MsPUBS050* | MS.gene76722 | chr4.1:70967479-70969590 | II | 0 | 703 |
| *MsPUBS051* | MS.gene004048 | chr4.2:41379272-41383753 | II | 3 | 654 |
| *MsPUBS052* | MS.gene006973 | chr4.2:25389200-25391742 | II | 3 | 652 |
| *MsPUBS053* | MS.gene04743 | chr4.2:2320391-2327036 | II | 12 | 1256 |
| *MsPUBS054* | MS.gene09196 | chr4.2:14889678-14893638 | II | 4 | 767 |
| *MsPUBS055* | MS.gene40168 | chr4.2:20639956-20641083 | II | 1 | 267 |
| *MsPUBS056* | MS.gene42604 | chr4.2:41401791-41406271 | II | 3 | 654 |
| *MsPUBS057* | MS.gene56818 | chr4.2:25309694-25312236 | II | 3 | 652 |
| *MsPUBS058* | MS.gene82233 | chr4.2:75070112-75072223 | II | 0 | 703 |
| *MsPUBS059* | MS.gene015765 | chr4.3:2269400-2277343 | II | 14 | 1337 |
| *MsPUBS060* | MS.gene08246 | chr4.3:16598203-16602161 | II | 4 | 766 |
| *MsPUBS061* | MS.gene28695 | chr4.3:22375430-22376557 | II | 0 | 375 |
| *MsPUBS062* | MS.gene77048 | chr4.3:70750910-70753021 | II | 0 | 703 |
| *MsPUBS063* | MS.gene021110 | chr4.4:42278482-42282954 | II | 3 | 662 |
| *MsPUBS064* | MS.gene023397 | chr4.4:15954792-15959333 | II | 4 | 767 |
| *MsPUBS065* | MS.gene028584 | chr4.4:22216945-22218018 | II | 0 | 357 |
| *MsPUBS066* | MS.gene05900 | chr4.4:3171729-3179694 | II | 14 | 1337 |
| *MsPUBS067* | MS.gene48822 | chr4.4:73893385-73895196 | II | 0 | 603 |
| *MsPUBS068* | MS.gene047796 | chr5.1:11962400-11965631 | II | 5 | 669 |
| *MsPUBS069* | MS.gene20298 | chr5.1:7969438-7975569 | II | 10 | 592 |
| *MsPUBS070* | MS.gene29673 | chr5.1:65215991-65217214 | II | 0 | 407 |
| *MsPUBS071* | MS.gene010520 | chr5.2:11816355-11819586 | II | 5 | 669 |
| *MsPUBS072* | MS.gene010524 | chr5.2:11824298-11827003 | II | 2 | 710 |
| *MsPUBS073* | MS.gene027623 | chr5.2:24364218-24366287 | II | 0 | 689 |
| *MsPUBS074* | MS.gene09600 | chr5.2:7319303-7325434 | II | 10 | 592 |
| *MsPUBS075* | MS.gene76078 | chr5.2:71037615-71039699 | II | 1 | 663 |
| *MsPUBS076* | MS.gene02444 | chr5.3:11867261-11870569 | II | 4 | 767 |
| *MsPUBS077* | MS.gene43541 | chr5.3:8435277-8441489 | II | 10 | 591 |
| *MsPUBS078* | MS.gene45047 | chr5.3:23318234-23320303 | II | 0 | 689 |
| *MsPUBS079* | MS.gene46229 | chr5.3:67210453-67211676 | II | 0 | 407 |
| *MsPUBS080* | MS.gene015258 | chr5.4:9088555-9094792 | II | 10 | 592 |
| *MsPUBS081* | MS.gene59383 | chr5.4:12520794-12523839 | II | 4 | 765 |
| *MsPUBS082* | MS.gene80282 | chr5.4:24360324-24362393 | II | 0 | 689 |
| *MsPUBS083* | MS.gene94226 | chr5.4:65773609-65775099 | II | 1 | 465 |
| *MsPUBS084* | MS.gene57130 | chr6.2:4051958-4053544 | II | 0 | 528 |

**Table S1. (continued)**

| | | | | | |
|---|---|---|---|---|---|
| *MsPUBS085* | MS.gene20604 | chr6.3:4336896-4338482 | II | 0 | 528 |
| *MsPUBS086* | MS.gene018692 | chr7.1:10791690-10796512 | II | 4 | 803 |
| *MsPUBS087* | MS.gene025882 | chr7.1:610045-614761 | II | 3 | 1000 |
| *MsPUBS088* | MS.gene071317 | chr7.1:87897273-87902224 | II | 7 | 1074 |
| *MsPUBS089* | MS.gene65150 | chr7.1:42019320-42024777 | II | 4 | 634 |
| *MsPUBS090* | MS.gene020378 | chr7.2:13380397-13385218 | II | 4 | 802 |
| *MsPUBS091* | MS.gene024415 | chr7.2:679637-684352 | II | 3 | 1000 |
| *MsPUBS092* | MS.gene91750 | chr7.2:38052184-38057559 | II | 4 | 634 |
| *MsPUBS093* | MS.gene050669 | chr7.3:692234-696951 | II | 3 | 1000 |
| *MsPUBS094* | MS.gene23476 | chr7.3:14401950-14406770 | II | 4 | 802 |
| *MsPUBS095* | MS.gene64577 | chr7.3:45128061-45133623 | II | 4 | 634 |
| *MsPUBS096* | MS.gene85423 | chr7.3:28796191-28799338 | II | 3 | 647 |
| *MsPUBS097* | MS.gene023937 | chr7.4:27923833-27927101 | II | 3 | 643 |
| *MsPUBS098* | MS.gene023939 | chr7.4:27888918-27892200 | II | 3 | 647 |
| *MsPUBS099* | MS.gene058531 | chr7.4:27899571-27902282 | II | 3 | 450 |
| *MsPUBS100* | MS.gene058533 | chr7.4:27953152-27956335 | II | 8 | 567 |
| *MsPUBS101* | MS.gene22720 | chr7.4:746458-751175 | II | 3 | 1000 |
| *MsPUBS102* | MS.gene54372 | chr7.4:93743030-93747982 | II | 8 | 1063 |
| *MsPUBS103* | MS.gene90349 | chr7.4:43999192-44004569 | II | 4 | 634 |
| *MsPUBS104* | MS.gene96991 | chr7.4:12615230-12620053 | II | 4 | 803 |
| *MsPUBS105* | MS.gene060520 | chr8.1:75196414-75200260 | II | 3 | 1006 |
| *MsPUBS106* | MS.gene56926 | chr8.1:18651124-18653199 | II | 0 | 691 |
| *MsPUBS107* | MS.gene76415 | chr8.1:30510561-30514458 | II | 5 | 702 |
| *MsPUBS108* | MS.gene012010 | chr8.2:28262731-28266636 | II | 4 | 760 |
| *MsPUBS109* | MS.gene067300 | chr8.2:70798560-70802527 | II | 3 | 1006 |
| *MsPUBS110* | MS.gene60356 | chr8.2:19237750-19239825 | II | 0 | 691 |
| *MsPUBS111* | MS.gene48390 | chr8.3:17062886-17064961 | II | 0 | 691 |
| *MsPUBS112* | MS.gene84731 | chr8.3:67182819-67186583 | II | 3 | 1006 |
| *MsPUBS113* | MS.gene87463 | chr8.3:26901454-26905385 | II | 4 | 759 |
| *MsPUBS114* | MS.gene033231 | chr8.4:20595502-20597577 | II | 0 | 691 |
| *MsPUBS115* | MS.gene84864 | chr8.4:69763832-69767596 | II | 3 | 1006 |
| *MsPUBS116* | MS.gene034749 | chr1.1:44081963-44083222 | III | 0 | 419 |
| *MsPUBS117* | MS.gene034750 | chr1.1:44095684-44097006 | III | 0 | 440 |
| *MsPUBS118* | MS.gene034751 | chr1.1:44110121-44111446 | III | 0 | 441 |
| *MsPUBS119* | MS.gene034753 | chr1.1:44180648-44181973 | III | 0 | 441 |
| *MsPUBS120* | MS.gene034756 | chr1.1:44240154-44241374 | III | 0 | 406 |
| *MsPUBS121* | MS.gene24493 | chr1.1:4825304-4826521 | III | 0 | 405 |
| *MsPUBS122* | MS.gene00522 | chr1.2:5249383-5250600 | III | 0 | 405 |
| *MsPUBS123* | MS.gene071765 | chr1.2:44977909-44978593 | III | 0 | 228 |
| *MsPUBS124* | MS.gene41035 | chr1.2:44953702-44954817 | III | 1 | 348 |
| *MsPUBS125* | MS.gene41036 | chr1.2:44997165-44998280 | III | 0 | 372 |
| *MsPUBS126* | MS.gene41211 | chr1.2:45035245-45035929 | III | 0 | 228 |
| *MsPUBS127* | MS.gene45454 | chr1.2:44802847-44804172 | III | 0 | 441 |

**Table S1. (continued)**

| | | | | | |
|---|---|---|---|---|---|
| *MsPUBS128* | MS.gene45455 | chr1.2:44826183-44827496 | III | 0 | 437 |
| *MsPUBS129* | MS.gene45456 | chr1.2:44857169-44858494 | III | 0 | 441 |
| *MsPUBS130* | MS.gene051676 | chr1.3:4957703-4959007 | III | 0 | 434 |
| *MsPUBS131* | MS.gene41031 | chr1.3:41633942-41635210 | III | 0 | 422 |
| *MsPUBS132* | MS.gene41032 | chr1.3:41648532-41649857 | III | 0 | 441 |
| *MsPUBS133* | MS.gene41033 | chr1.3:41714053-41715381 | III | 0 | 442 |
| *MsPUBS134* | MS.gene066125 | chr1.4:47244913-47246133 | III | 0 | 406 |
| *MsPUBS135* | MS.gene066129 | chr1.4:47207921-47209246 | III | 0 | 441 |
| *MsPUBS136* | MS.gene066130 | chr1.4:47182366-47183691 | III | 0 | 441 |
| *MsPUBS137* | MS.gene066131 | chr1.4:47168012-47169273 | III | 0 | 420 |
| *MsPUBS138* | MS.gene66476 | chr1.4:5134001-5135305 | III | 0 | 434 |
| *MsPUBS139* | MS.gene45494 | chr2.1:6792886-6797859 | III | 6 | 753 |
| *MsPUBS140* | MS.gene30960 | chr2.2:5269917-5274887 | III | 6 | 753 |
| *MsPUBS141* | MS.gene052119 | chr2.3:5814508-5819021 | III | 6 | 753 |
| *MsPUBS142* | MS.gene31153 | chr2.4:6578193-6583164 | III | 6 | 753 |
| *MsPUBS143* | MS.gene008306 | chr3.1:54542523-54543821 | III | 0 | 432 |
| *MsPUBS144* | MS.gene049312 | chr3.1:76306638-76307888 | III | 0 | 416 |
| *MsPUBS145* | MS.gene054961 | chr3.1:76706761-76707978 | III | 0 | 405 |
| *MsPUBS146* | MS.gene061543 | chr3.2:43858114-43859434 | III | 1 | 332 |
| *MsPUBS147* | MS.gene061546 | chr3.2:60484761-60486080 | III | 0 | 439 |
| *MsPUBS148* | MS.gene06399 | chr3.2:80454217-80455434 | III | 0 | 405 |
| *MsPUBS149* | MS.gene06437 | chr3.2:79943753-79945003 | III | 0 | 416 |
| *MsPUBS150* | MS.gene049279 | chr3.3:79837047-79838264 | III | 0 | 405 |
| *MsPUBS151* | MS.gene04998 | chr3.3:58676709-58678028 | III | 0 | 439 |
| *MsPUBS152* | MS.gene30995 | chr3.3:79297637-79298887 | III | 0 | 416 |
| *MsPUBS153* | MS.gene013306 | chr3.4:87278736-87279953 | III | 0 | 405 |
| *MsPUBS154* | MS.gene013340 | chr3.4:86847567-86848817 | III | 0 | 416 |
| *MsPUBS155* | MS.gene048677 | chr3.4:66542456-66543775 | III | 0 | 439 |
| *MsPUBS156* | MS.gene61649 | chr4.1:47085586-47086773 | III | 0 | 395 |
| *MsPUBS157* | MS.gene61650 | chr4.1:46996952-46998139 | III | 0 | 395 |
| *MsPUBS158* | MS.gene70238 | chr4.1:8590069-8591328 | III | 0 | 419 |
| *MsPUBS159* | MS.gene58093 | chr4.2:9335916-9337175 | III | 0 | 419 |
| *MsPUBS160* | MS.gene66842 | chr4.2:51260083-51261144 | III | 0 | 354 |
| *MsPUBS161* | MS.gene96107 | chr4.2:51081844-51083031 | III | 0 | 395 |
| *MsPUBS162* | MS.gene96108 | chr4.2:50983487-50984674 | III | 0 | 395 |
| *MsPUBS163* | MS.gene030641 | chr4.3:8471688-8472944 | III | 0 | 418 |
| *MsPUBS164* | MS.gene66646 | chr4.3:49039245-49040432 | III | 0 | 395 |
| *MsPUBS165* | MS.gene66647 | chr4.3:49026371-49027558 | III | 0 | 395 |
| *MsPUBS166* | MS.gene66649 | chr4.3:48953441-48954628 | III | 0 | 395 |
| *MsPUBS167* | MS.gene044728 | chr4.4:51431551-51432738 | III | 0 | 395 |
| *MsPUBS168* | MS.gene28406 | chr4.4:8982534-8983793 | III | 0 | 419 |
| *MsPUBS169* | MS.gene20311 | chr5.1:7827755-7829110 | III | 0 | 451 |
| *MsPUBS170* | MS.gene017373 | chr5.2:66035662-66036990 | III | 0 | 442 |

**Table S1. (continued)**

| | | | | | |
|---|---|---|---|---|---|
| *MsPUBS171* | MS.gene09612 | chr5.2:7167411-7168766 | III | 0 | 451 |
| *MsPUBS172* | MS.gene064492 | chr5.3:62894817-62896145 | III | 0 | 442 |
| *MsPUBS173* | MS.gene43528 | chr5.3:8259025-8260380 | III | 0 | 451 |
| *MsPUBS174* | MS.gene015270 | chr5.4:8945750-8947105 | III | 0 | 451 |
| *MsPUBS175* | MS.gene26139 | chr5.4:57136265-57137593 | III | 0 | 442 |
| *MsPUBS176* | MS.gene042912 | chr6.1:64600650-64601966 | III | 0 | 438 |
| *MsPUBS177* | MS.gene93972 | chr6.2:73813191-73814507 | III | 0 | 438 |
| *MsPUBS178* | MS.gene022190 | chr6.3:67932781-67934097 | III | 0 | 438 |
| *MsPUBS179* | MS.gene72611 | chr6.4:48797053-48798369 | III | 0 | 438 |
| *MsPUBS180* | MS.gene023900 | chr7.1:25922090-25927494 | III | 8 | 885 |
| *MsPUBS181* | MS.gene025667 | chr7.1:6406666-6407928 | III | 0 | 420 |
| *MsPUBS182* | MS.gene007121 | chr7.2:7586739-7588001 | III | 0 | 420 |
| *MsPUBS183* | MS.gene27767 | chr7.2:28346478-28351880 | III | 8 | 885 |
| *MsPUBS184* | MS.gene51606 | chr7.2:28210328-28215690 | III | 8 | 885 |
| *MsPUBS185* | MS.gene26489 | chr7.3:7824010-7825272 | III | 0 | 420 |
| *MsPUBS186* | MS.gene97763 | chr7.3:81871503-81876494 | III | 7 | 1073 |
| *MsPUBS187* | MS.gene31152 | 15257:3022-7994 | III | 6 | 753 |
| *MsPUBS188* | MS.gene006267 | chr1.1:72117672-72125451 | IV | 7 | 715 |
| *MsPUBS189* | MS.gene34143 | chr1.2:75120093-75127644 | IV | 7 | 715 |
| *MsPUBS190* | MS.gene49114 | chr1.3:69029629-69037865 | IV | 7 | 712 |
| *MsPUBS191* | MS.gene061161 | chr1.4:77914822-77922596 | IV | 7 | 715 |
| *MsPUBS192* | MS.gene84344 | chr3.1:3828697-3833803 | IV | 8 | 797 |
| *MsPUBS193* | MS.gene84345 | chr3.1:3837663-3842482 | IV | 8 | 809 |
| *MsPUBS194* | MS.gene058376 | chr3.2:2530452-2535612 | IV | 8 | 796 |
| *MsPUBS195* | MS.gene058377 | chr3.2:2538266-2542823 | IV | 8 | 809 |
| *MsPUBS196* | MS.gene56216 | chr3.3:4062755-4067897 | IV | 8 | 796 |
| *MsPUBS197* | MS.gene56217 | chr3.3:4070003-4074734 | IV | 8 | 809 |
| *MsPUBS198* | MS.gene009413 | chr3.4:3499059-3504197 | IV | 8 | 796 |
| *MsPUBS199* | MS.gene009414 | chr3.4:3507021-3511811 | IV | 8 | 809 |
| *MsPUBS200* | MS.gene046436 | chr5.1:22012304-22017761 | IV | 8 | 809 |
| *MsPUBS201* | MS.gene027794 | chr5.2:22032026-22036898 | IV | 8 | 808 |
| *MsPUBS202* | MS.gene070689 | chr5.3:20654174-20659382 | IV | 8 | 808 |
| *MsPUBS203* | MS.gene069114 | chr5.4:22177458-22182347 | IV | 8 | 809 |
| *MsPUBS204* | MS.gene065220 | chr8.1:36427144-36430656 | IV | 7 | 779 |
| *MsPUBS205* | MS.gene008939 | chr8.2:34440957-34444481 | IV | 7 | 785 |
| *MsPUBS206* | MS.gene53517 | chr1.2:27709441-27719634 | VI | 17 | 522 |
| *MsPUBS207* | MS.gene053443 | chr1.3:26648382-26658603 | VI | 17 | 522 |
| *MsPUBS208* | MS.gene022812 | chr8.1:84992775-84996427 | VII | 6 | 238 |
| *MsPUBS209* | MS.gene37050 | chr8.2:80246730-80250733 | VII | 7 | 277 |
| *MsPUBS210* | MS.gene020135 | chr8.3:77666547-77670281 | VII | 7 | 277 |
