# Supplementary material for: Genome-Wide Identification and Expression Analysis of the Alfalfa (Medicago sativa L.) U-Box Gene Family in Response to Abiotic Stresses
Source: Int J Mol Sci. 2024 Nov 17;25(22):12324. doi: 10.3390/ijms252212324 (PMC11595061; doi:10.3390/ijms252212324)
Supplement: Supplementary file 1 [file ijms-25-12324-s001.zip › Table S2.pdf]

Table S2. Primers used for qRT-PCR analysis of the MsPUBs

**Name**	**Forward primer sequences (5'→3')**	**Reverse primer sequences (5'→3')**
MsPUBS067	ACAGACTTTAACGGTGTTAGG	ATTCTCCGGCGAAGATAGATT
MsPUBS106	CAATCGATCGAAGAGTGGAG	TCACTTCTCTGTCATTGGACT
MsPUBS110	ATGTGACTGAGCTTGTGTTC	CAACGTTGAATTCTTCGCAA
MsPUBS111	GTTGGGTAGAAACCGAACAA	AATTGCTAGGTCATCGGTTG
MsPUBS130	CTAGGCCCAGGAGTAAAACT	AACTTGCTTCTCTAAGGGGT
MsPUBS155	CTGGTGTTGTTGGTGAGAAA	ACAAGTACTTCTGAGAAAACCA
MsPUBS158	GATGAAAGACCCCGTTACAC	ATACGTGGAGTTGGGATTCT
MsPUBS163	ACTCTTCGCCATTTGATTGA	GCAAGATTTTCTGAAAGAGCAA
MsPUBS177	GAAGGAGTTGAAGAGGCTTG	CAGAAGCAACAATGACAGGA
MsPUBS185	ACACTGATCTAACACCAAACC	TCAAGCATTTAAGCTGCACA
MsPUBS210	CAGTTGGAGGCTGTAGAAAG	TTCATGGACTGCTTCCCTAA
GAPDH	TTTTTCAGCCATGGGCAA	TCATCGTTTTTCCACTGTCC
