# Supplementary figures and images for: Genome-Wide Identification and Expression Analysis of the Alfalfa (Medicago sativa L.) U-Box Gene Family in Response to Abiotic Stresses
Source: Int J Mol Sci. 2024 Nov 17;25(22):12324. doi: 10.3390/ijms252212324 (PMC11595061; doi:10.3390/ijms252212324)

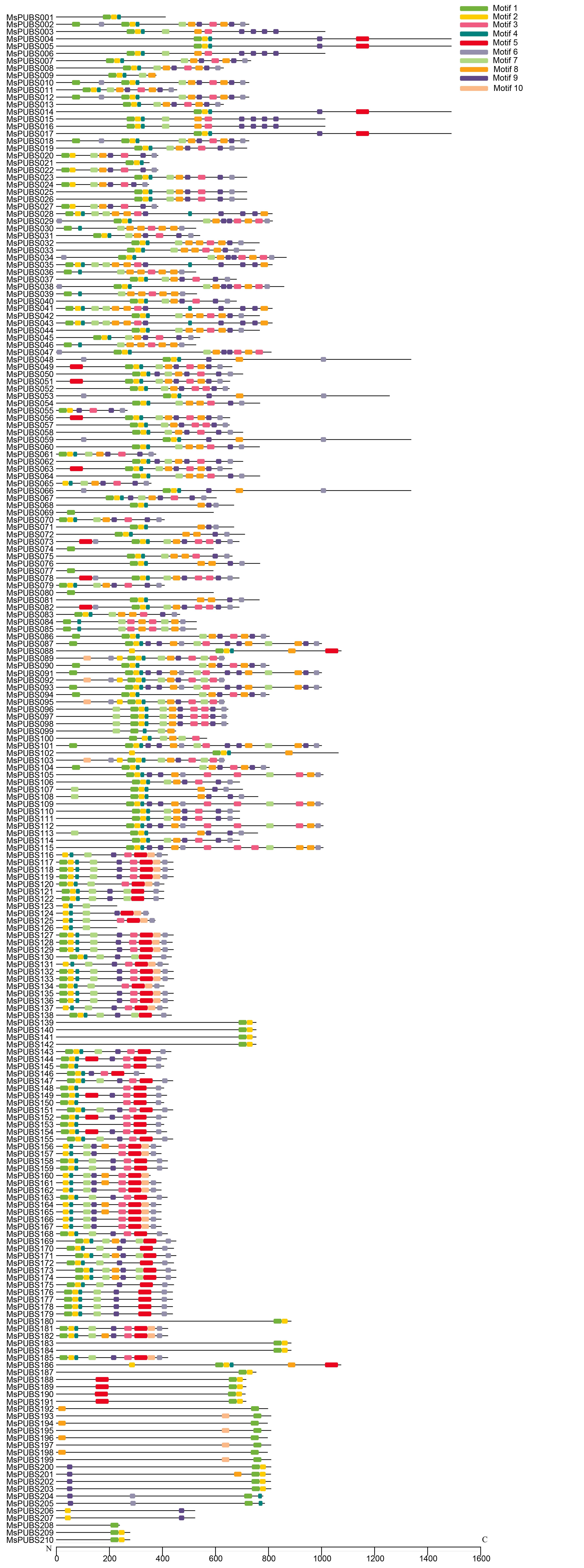

Supplement: Supplementary file 1 [file ijms-25-12324-s001.zip › Figure S1.jpg]

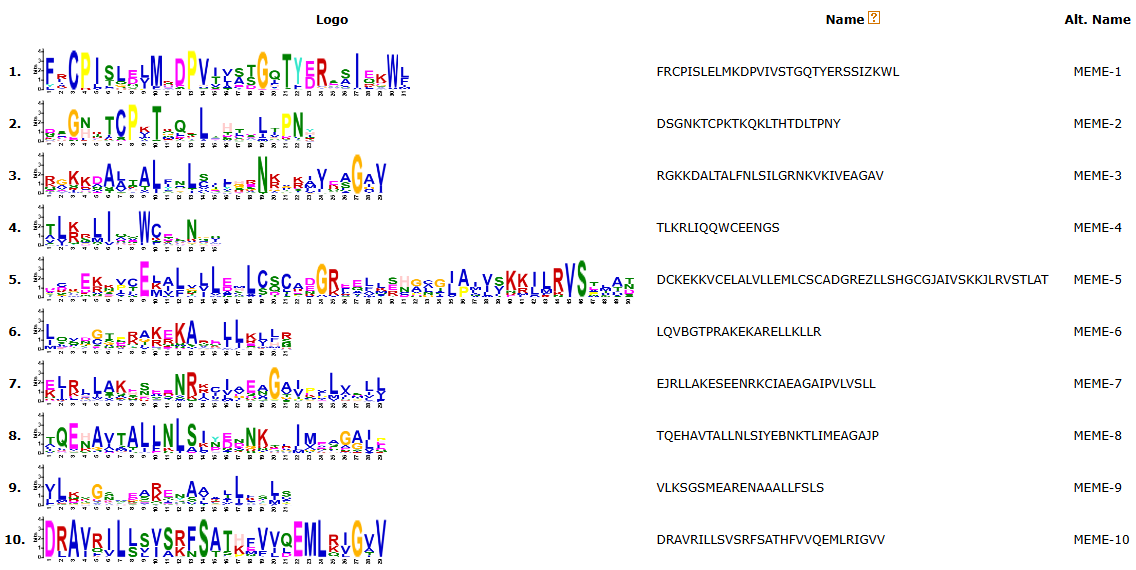

Supplement: Supplementary file 1 [file ijms-25-12324-s001.zip › Figure S2.png]

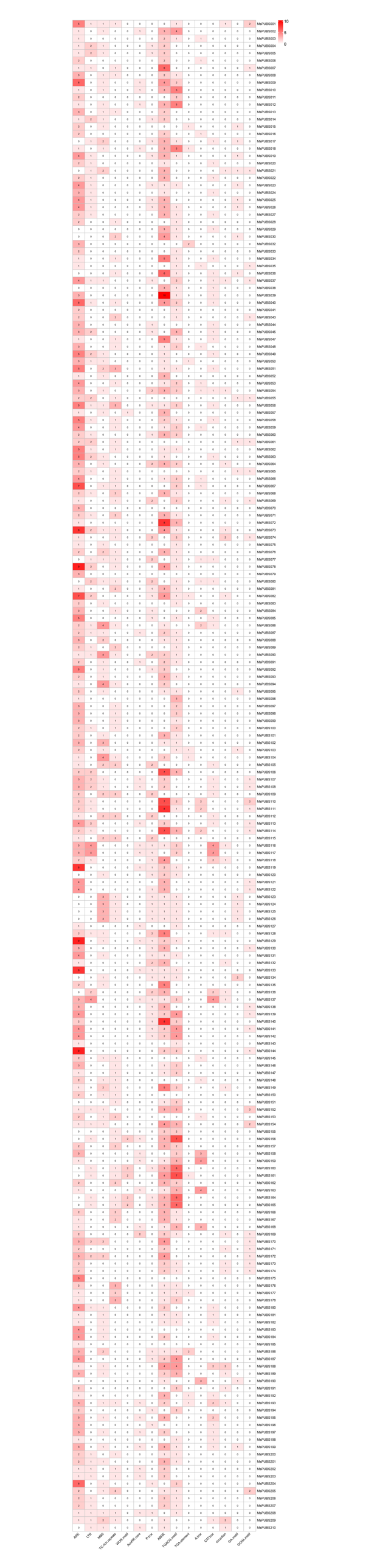

Supplement: Supplementary file 1 [file ijms-25-12324-s001.zip › Figure S3.jpg]
